# Comparison between the induced membrane technique and distraction osteogenesis in treating segmental bone defects: An experimental study in a rat model

**DOI:** 10.1371/journal.pone.0226839

**Published:** 2019-12-20

**Authors:** Zhen Shen, Haixiong Lin, Guoqian Chen, Yan Zhang, Zige Li, Ding Li, Lei Xie, Yue Li, Feng Huang, Ziwei Jiang

**Affiliations:** 1 First Clinical Medical College, Guangzhou University of Chinese Medicine, Guangzhou, Guangdong, China; 2 Department of Orthopaedics, First Affiliated Hospital of Hunan Traditional Chinese Medical College, Zhuzhou, Hunan, China; 3 Fifth Clinical Medical College, Guangzhou University of Chinese Medicine, Guangzhou, Guangdong, China; 4 Tropical Medicine Institute, Guangzhou University of Chinese Medicine, Guangzhou, Guangdong, China; 5 Department of Orthopaedics, First Affiliated Hospital of Guangzhou University of Chinese Medicine, Guangzhou, Guangdong, China; Università degli Studi della Campania, ITALY

## Abstract

Previous studies have suggested that treatment plans for segmental bone defects (SBDs) are affected by the bone defect sizes. If the selected treatment was not the most appropriate, it would not contribute to bone healing, but increase complications. The induced membrane technique (IM) and distraction osteogenesis (DO) have been proved to be effective in treating SBDs. However, the differences between the two in therapeutic effects on SBDs with different sizes are still unclear. Thus, we aimed to observe the effects of IM and DO on different sizes of SBDs and to further determine what method is more appropriate for what defect size. Rat models of 4-, 6-and 8-mm mid-diaphyseal defects using IM and DO techniques were established. X-rays, micro-CT, histological and immunohistochemical examinations were performed to assess bone repair. Faster bone formation rate, shorter treatment duration, higher expressions of OPN and OCN and higher parameters of bone properties including bone mineral density (BMD), bone volume/total tissue volume (BV/TV), mineral apposition rate (MAR) and mineral surface/bone surface (MS/BS) were found in 4-mm SBDs treated with DO than in those with IM treatment. However, the results were reversed and IM outperformed DO in bone repair capacity for 8-mm SBDs, while no significant difference emerges in the case of 6-mm SBDs. This study suggests that the therapeutic effects of IM and DO may be subjected to sizes of bone defects and the best treatment size of defects is different between the two. For small-sized SBDs, DO may be more suitable and efficient than IM, but IM has advantages over DO for over-sized SBDs, while DO and IM show similar bone repair capability in moderate-sized SBDs, which would offer a new insight into how to choose DO and IM for SBDs in clinical practice and provide references for further clinical research.

## Introduction

Despite technological advances, Management of segmental bone defects (SBDs) currently is still a tremendous problem in clinical practice. It is reported that SBDs affect over two million people worldwide with an economic burden of US $3 billion every year and cause significant pain and disability including limb deformity and dysfunction, in addition to lengthy treatment duration, substantial complication rates and high health-care costs [1, 2]. As is well known, small defects, 2 cm in length recommended as the maximum size of a segmental diaphyseal tibial defect[3, 4], may be managed with autologous cancellous bone grafting alone[5], while larger segmental bone defects, especially in excess of 4–5 cm[6, 7], typically require bone transport via distraction osteogenesis (DO) or bone graft through the induced membrane (IM)[8, 9], which means the choice of therapeutic strategies is affected by the size of the bone defect. Since the choice of treatment is subjected to the size of the bone defect, is it possible that unsatisfactory treatment effects could be due to the reason that the treatment option is not the most appropriate in the face of bone defects of different sizes?

The IM technique for the treatment of SBDs consists of a 2-stage procedure [10, 11]. In the first step, the defect site is stabilized with external or internal fixation, and a cement spacer made of polymethylmethacrylate (PMMA) is inserted into the bone defect gap. After soft tissue closure, over a period of 2–6 weeks, a foreign-body reaction induces and forms a membrane that encapsulates the cement spacer. In the second step, the induced membrane is opened, the PMMA spacer is removed and the resulting cavity is filled with autologous bone. In contrast, DO, a unique and effective technique for bone regeneration, is composed of three sequential phases: the latency phase after osteotomy and application of the external fixator; the distraction phase during which the osteotomized bone ends were separated by gradual and continuous distraction; and the consolidation stage until the newly formed bone is mechanically strong enough [12, 13].

Since the concepts of DO and IM were introduced by Ilizarov [14] and Masquelet [10] in 1969 and 2000, respectively, DO and IM have been extensively studied and become widely used for SBDs in clinical practice [15–17]. Various types of clinical reports including prospective[18] or retrospective studies[19, 20] and meta-analyses[21, 22] indicated that the IM and DO techniques were effective in treating SBDs. However, the differences between the two in therapeutic effects on SBDs of different sizes have not yet been fully elucidated. For one thing, previous comparisons [23, 24] between IM and DO were mainly confined to clinical research, whereas original studies of basic research were rather few. For another, more attention was mainly paid to the soft tissue condition, infection and cost. Even the surgeon’s preferences and experiences could exert influence on the choice of the treatment methods [25], but the defect size appearing to be the most fundamental aspect was barely taken into consideration. Up to now, there are few such studies on basic research involving both DO and IM and almost no direct comparisons of DO to IM from the perspective of bone defect sizes. As far as we know, research regarding the effects of different defect sizes on selection from DO and IM in animal models of SBDs were not published before. As a result, it is still not clear how to choose the two techniques in the case of bone defects over 5 cm, not taking into account other factors like infection, skin coverage or treatment cost. Further speaking, it still remains unknown that in what size of defects IM could achieve better results, while what size of defects is more suitable for DO.

Thus, the purpose of the present study was to observe whether differences would emerge in the therapeutic effects of IM and DO on SBDs of different sizes and to further determine what method is more appropriate for what defect size.

## Materials and methods

### Ethics statement

All animal care and experimental procedures were approved by the Institutional Animal Ethics Committee of the First Affiliated Hospital of Guangzhou University of Chinese Medicine (ethical approval number: TCMF1-2018002).

### Animals

A total of fifty-four adult male Sprague-Dawley (SD) rats with similar age (20 weeks) and weight (380–420 g) were obtained from Guangdong Medical Lab Animal Research Center. The rats were kept in specific pathogen-free (SPF)-class housing in the laboratory with standard conditions at 24 °C under 12:12 h light-dark cycle and fed with a standard diet. Based on the sizes of bone defects, all rats were randomly assigned to one of the three defect groups: 4 mm, 6 mm or 8 mm (18 rats per group).

### Creation of segmental bone defect (SBD) model by the internal plate and external fixator

**Fig 1:**
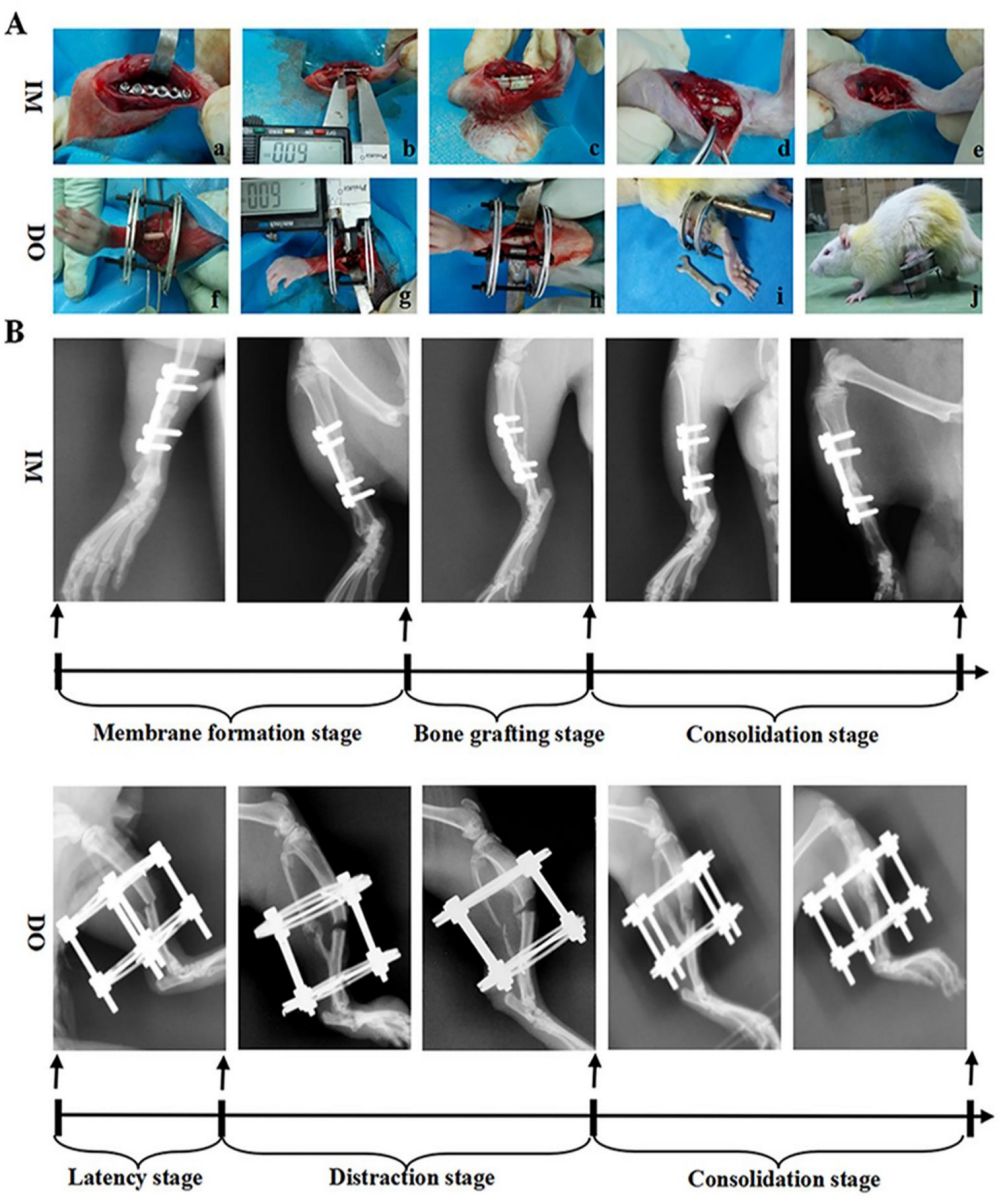
Animal models of SBD in rats. (A) Flowcharts of surgical procedures of SBD models by the internal plate and external fixator. (a): tibia exposure and plate fixation; (b): osteotomy; (c): bone cement implantation; (d): induced membrane formation; (e): bone grafting; (f): tibia exposure and external fixator fixation; (g): osteotomy; (h): shortening and aligning osteotomy ends; (i): distraction procedure; (j): consolidation stage. (B) Protocols of IM and DO shown by X-ray.

After 2 weeks of acclimatization to the housing environment, the rat SBD model was established according to previous protocols [26, 27], with minor modifications. In brief, after anesthetization with intraperitoneal pen-tobarbital (3 mg/100 g, Sigma, St. Louis, MO, USA), a longitudinal incision was made in the skin distal to the tibia crest and the bone was exposed. Meanwhile, surgical scissors were used to snip the fibula. Then a custom-made stainless-steel mini-plate was applied to the anterior aspect of the left tibia shaft and secured with cortical screws (plate: 15 mm long, 6-hole, screws: 2.0-mm diameter, Baokang, Zhangjiagang, China) (Fig 1a), while a custom-made circle external distraction device was assembled and fixed to the right tibia by four 27-gauge stainless steel needles (Baokang, Zhangjiagang, China) (Fig 1f). After stabilization, transverse corticotomies using a Gigli saw (Baokang, Zhangjiagang, China) were performed to create 4-, 6- and 8-mm long mid-diaphyseal defects on bilateral tibias, respectively (Fig 1b and 1g). Following surgery, rats were allowed to eat and drink ad libitum. Antibiotic (amoxicillin 1.5 mg/100g weight) and buprenorphine (1.0 mg/kg weight) were administered intraperitoneally for following 3 days, respectively.

Subsequent to the construction of the above-mentioned SBDs, bone defects of left tibias were firstly filled with the polymethylmethacrylate (PMMA) spacer (Fig 1c). Four weeks following the first procedure, a membrane was formed (Fig 1d) and then removal of the spacer and implantation of autologous corticocancellous bone harvested from the vertebrae of the tail were performed (Fig 1e). Of note, soft tissue covering the surface of vertebrae was removed and then the extracted vertebrae were ground to fill the defect gap under sterile conditions. Grafted bone mass amounts to approximately two caudal vertebrae for 4-mm defect area, three caudal vertebrae for 6-mm defect area and four caudal vertebrae for 8-mm defect area. In addition, the time point of four weeks was chosen for this study because many characteristics of induced membranes peak around this time [28] and it is the current clinical recommendation [29]. By contrast, the two corticotomy surfaces of right tibias were shortened and brought into close apposition (Fig 1h), which was followed by a 7-day latency period. Then the distraction procedure was initiated at a rate of 0.1 mm per 12 hours until the length of osteotomy was restored (Fig 1i). Before the rats were sacrificed, bone segments were maintained the position with external device throughout this study (Fig 1j). During the whole construction of the model, rats were monitored twice a day and no animal became severely infected, ill or died at any time prior to the experimental endpoint. Rats were euthanized by an overdose of intraperitoneal pen-tobarbital.

### Radiographic assessment

The consecutive X-ray photographs were obtained at 8, 12, and 14 weeks after surgery (n = 9 per group, n = 3 per time point) to show the dynamic healing process of bone defects. Meanwhile, radiographs were evaluated by 3 independent orthopedic surgeons who were blind to the therapies and groups. The results were scored using the radiographic scoring system described by Lane et al. [30]. Briefly, The following three aspects including bone formation, persistence of fracture line, and remodeling were assessed readily in living specimens at 8, 12 and 14 weeks after surgery, respectively, by the radiographic system shown in Table 1. All tests were repeated with three specimens.

**Table 1:** Radiographic scoring.

Points	
**Bone formation**	
No evidence of bone formation	**0**
Bone formation occupying 25% of defect	**1**
Bone formation occupying 50% of defect	**2**
Bone formation occupying 75% of defect	**3**
Full gap bone formation	**4**
**Union**	
Full fracture line	**0**
Partial fracture line	**2**
Absent fracture line	**4**
**Remodeling**	
No evidence of remodeling	**0**
Remodeling of intramedullary canal	**2**
Full remodeling of cortex	**4**

### Micro-CT analysis

After X-ray examination, tibias were immediately harvested and fixed in 10% neutral buffered for approximately 48 hours. Then, samples (n = 3 per group) were scanned with micro-CT (SkyScan 1076, Kontich, Belgium) at a resolution of 20 μm (70kV and 130μA radiation source with 0.5 mm aluminum filter). The bone mineral density (BMD) and bone tissue volume/ total tissue volume (BV/TV) inside the defect gaps were determined using Scanco software. All tests were repeated with three specimens.

### Histological and immunohistochemical observation

For the reason that many biological characteristics of induced membranes peak around 4 weeks after surgery [28, 29], the formed membrane samples (n = 3 per group) were harvested at 4 weeks after surgery and then decalcified, dehydrated, embedded in paraffin and sectioned into 5 mm thick sections. Five sequential sections per rat in each group were stained with hematoxylin-eosin (HE). We counted the numbers of vessels in the four random visual fields of per section. Additionally, after the micro-CT scanning, the tibia samples above-mentioned were processed for histology, which were decalcified in 10% EDTA for four weeks and sectioned into 5 mm thick sections for HE and Masson’s trichrome (Masson’s) staining. Meanwhile, nondecalcified tibia samples (n = 3 per group) were also harvested at 14 weeks after surgery, which were stained with McNeal’s tetrachrome, toluidine blue O, and basic fuchsin (McNeal’s), as reported previously, with minor modifications [31]. Briefly, the harvested samples were fixed, dehydrated, and embedded in methylmethacrylate. Sections of 600 μm in thickness were produced using a microtome (Leica, RM2255, Germany). The undecalcified sections were polished down to 100 μm thickness and then stained with McNeal’s tetrachrome, toluidine blue O, and basic fuchsin. Mineralized bone tissue was stained pink and unmineralized tissue was light blue. All tests were repeated with three specimens.

Immunohistochemistry staining for osteopontin (OPN), osteocalcin (OCN), CD31 and VEGF was performed to detect mature osteoblasts and vessels. In brief, the sections embedded in paraffin were dewaxed, rehydrated and treated with antigen retrieval. Then the sections were incubated with the primary antibodies to rabbit osteopontin (OPN, Abcam, UK 1:200, abb448), osteocalcin (OCN, Santa Cruz, USA 1:100, sc30045), CD31 (Abcam, UK 1:200, ab64543) and VEGF (Abcam, UK 1:100, = ab46154) overnight at 4°C. Subsequently, the sections were incubated with the HRP-conjugated secondary antibody (Santa Cruz Biotechnology, Dallas, TX, USA), followed by counterstaining with hematoxylin. Images were saved using Image-Pro Plus software, version 6.0 (Media Cybernetics, Rockville, MD, USA), and the positive stained cell numbers and area in five random fields of the bone regeneration zone in three random sections from each specimen were counted and analyzed.

### Bone dynamic histomorphology

Three rats per group were intraperitoneally injected with calcein (20 mg/kg of body weight, Sigma, USA), dissolved in 2% sodium bicarbonate solution at 4 weeks after surgery and 3 days before sacrifice, separately. Rats were sacrificed at the 14th week and the specimens were fixed in 70% ethanol, dehydrated in acetone and embedded in methylmethacrylate. Sagittal sections of tibia in 150 μm were cut using a microtome (Leica, RM2255, Germany) and were subsequently ground and polished to a final thickness of about 10 mm for fluorescence labeling observation under confocal laser scanning microscope (Leica image analysis system, Q500MC). Mineral apposition rate (MAR) and mineral surface versus bone surface (MS/BS) were calculated and analyzed. All tests were repeated with three specimens.

### Microfil perfusion

Three rats per group were euthanized and perfused with Microfil (Microfil MV-122, Flow Tech; Carver, MA, USA) for angiography assay at 4 weeks postoperatively. Briefly, the rib cage was opened after anesthetization, the descending aorta was clamped, and the inferior vena cava was incised. Then the vasculature was flushed with 0.9% normal saline containing heparin sodium (100 U/mL) and 20 ml of Microfil were respectively perfused into the left ventricle with an angiocatheter. Subsequently, the rats were stored at 4°C overnight to ensure polymerization of the contrast agent and then decalcified in 10% ethylenediaminetetraacetic acid (EDTA) (Sigma, US) for four weeks. Images were obtained by micro-CT and the vessel volume within the membranes surrounding the bone defect region was evaluated.

### Statistical analyses

All data were presented as means ± standard deviations. Differences among groups were assessed by one-way analysis of variance (ANOVA) and paired t-tests were conducted for comparisons between two methods in one group. *P* < 0.05 was considered to be statistically significant. GraphPad Prism software (Version 6.01, La Jolla, CA, USA) was used for all statistical analyses.

## Results

### X-ray evaluation

**Fig 2:**
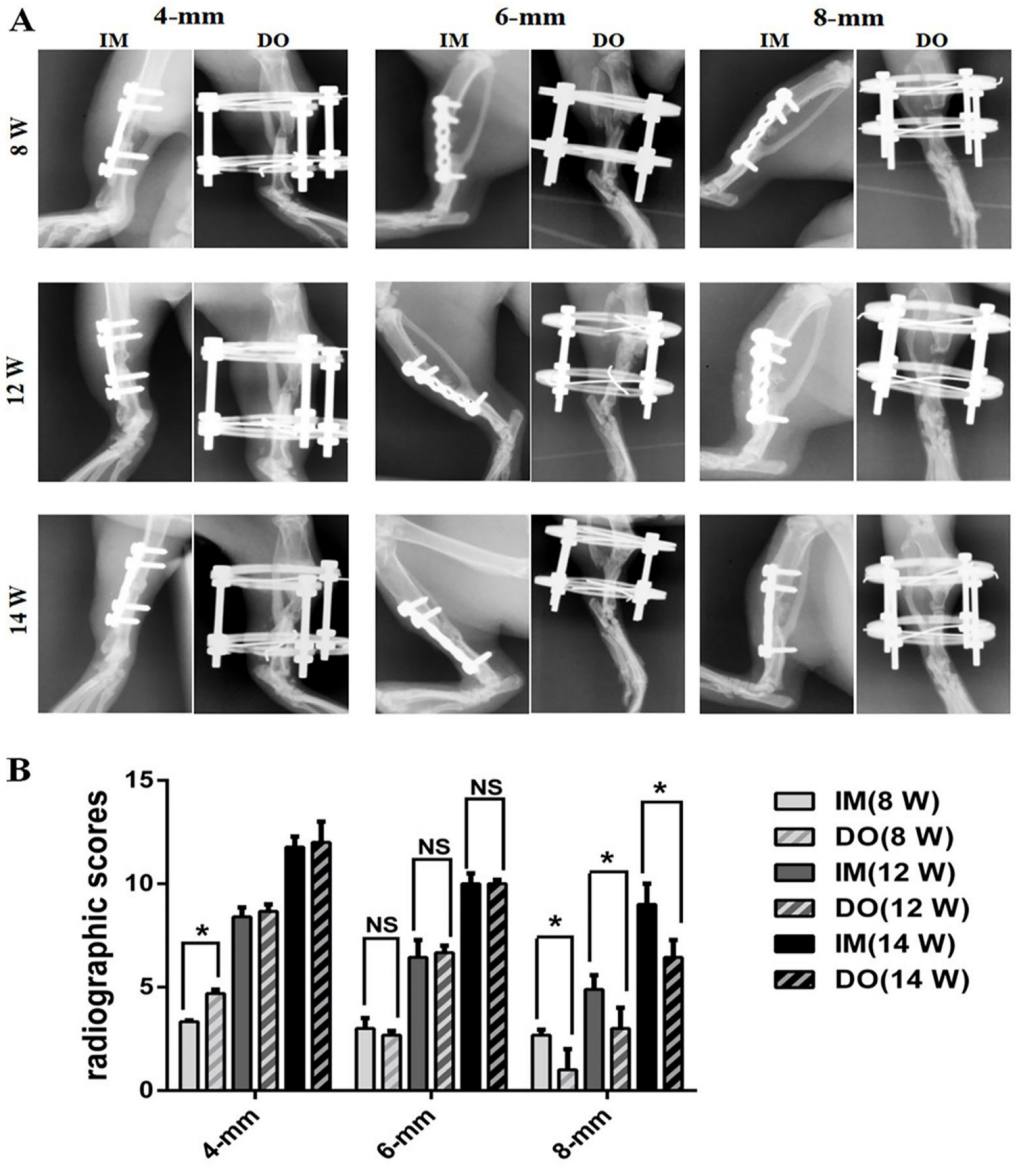
Radiological evaluation of bone repair in 4-,6- and 8-mm groups at 8, 12 and 14 weeks after surgery, respectively. (A) Series of representative radiographs of bilateral tibias treated with IM and DO. (B) Quantitative analysis of radiological scores. Significant difference is present when *P*<0.05 and displayed with an asterisk; NS: not significant.

X-ray images showed bone defects in each group healed slowly with time during the repair process and no nonunion occurred (Fig 2). But the time to bony connection in per group increased with the extension of the defect size. As presented in Fig 2A, at 8 weeks after surgery, only 4-mm group achieved partial bony union, while no radiographical defect bridging occurred in 6- and 8-mm groups. Additionally, in 4-mm group, DO resulted in much more mineralized callus formation and achieved earlier radiographical union than IM. However, in 6-mm group, DO and IM seemed to possess the similar rate and mass of bone formation, while in 8-mm group, the results were reversed and IM outperformed DO in mineralized callus formation. Descriptive statistical analysis of the radiological scores (Fig 2B) demonstrated that classification scores increased with time but decreased with defect size for each group. Interestingly, radiological scores of DO (4.67±0.58) were higher than those of IM (3.33±0.58) in 4-mm group at 8 weeks post operation, almost equal to those of IM in 6-mm group (week 8: 2.87±0.58 vs 3.00±1.00; week 12: 7.00±1.00 vs 6.67±1.15; week 14: 9.33±0.58 vs 9.67±0.58, respectively), but lower than those of IM (week 8: 1.67±1.15 vs 2.67±0.58; week 12: 3.00±1.00 vs 4.67±0.58; week 14: 7.00±1.00 vs 9.00±1.00, respectively) in 8-mm group at 8, 12 and 14 weeks post operation, respectively (Fig 2B).

### Micro-CT evaluation

**Fig 3:**
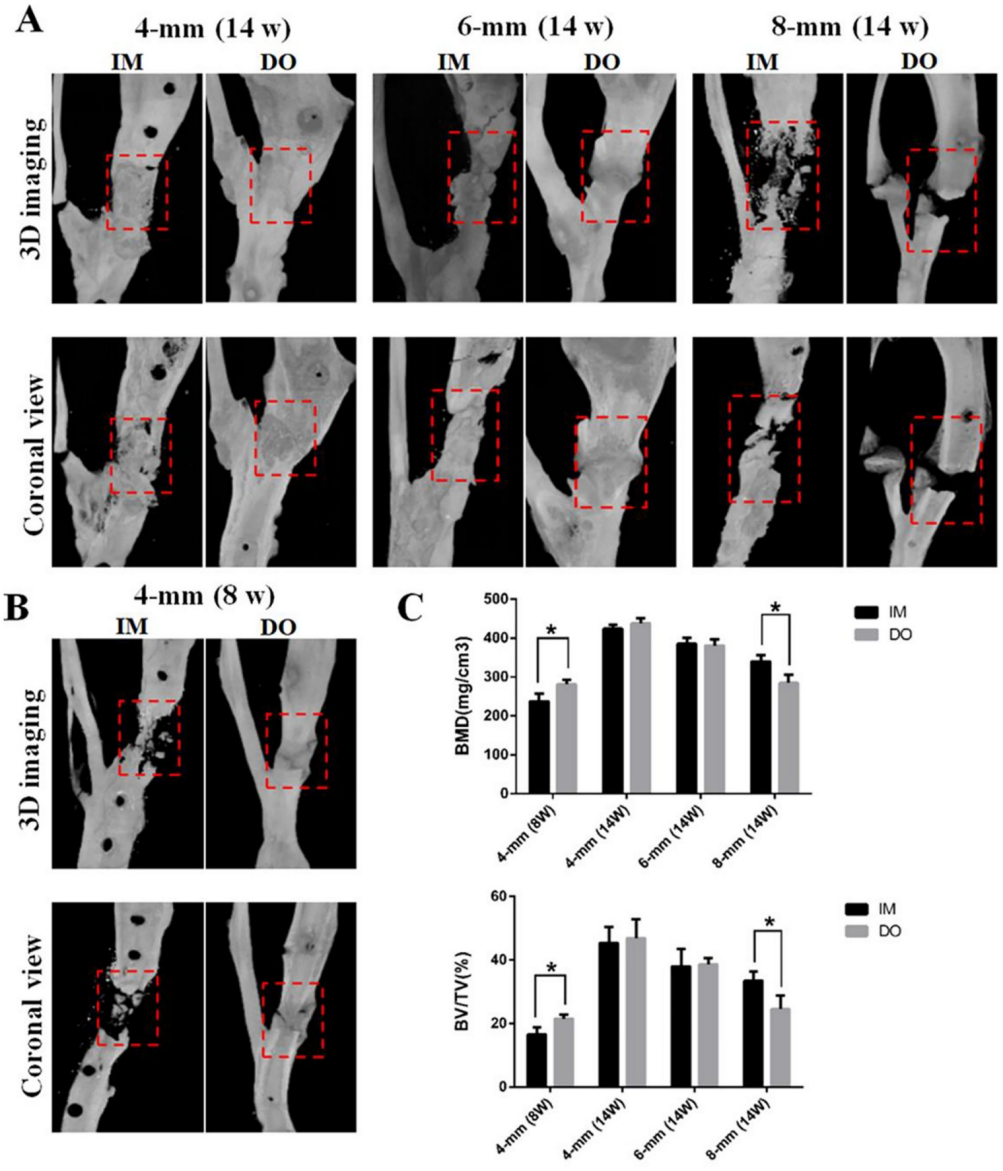
Representative micro-CT images of bone repair in the three groups. (A) Three-(top panel) and two-dimensional (lower panel) reconstructed images of bone defects at 14 weeks after surgery. Red dotted boxes indicate region of interest (ROI), representing bone defect areas. (B) Three- (left panel) and two-dimensional (right panel) reconstructed images of bone defects in 4-mm group at 8 weeks post-operation. (C) Quantification of bone mineral density (BMD), bone tissue volume/total tissue volume and (BV/TV) insides defect regions. (*P< 0.05; NS: not significant).

Similar to the manifestation of X-ray photography, in 4-mm group, DO led to more bone formation than IM as early as 8 weeks postoperatively, even though both achieved almost complete defect healing at 14 weeks postoperatively (Fig 3A and 3B). However, in 8-mm group, the results were reversed and IM outperformed DO in newly-formed bone, whereas no obvious difference was found in the bone formation between DO and IM in 6-mm group (Fig 3C). Quantitative analysis showed that BMD of the defect area treated with DO (285.71±14.49) was higher than that of IM-treated defect area (242.86±20.92) as early as 8 weeks post operation for the 4-mm group, but no difference showed at 14 weeks (432.58±13.94 vs 414.19±10.61). Likewise, the result in 8-mm group was opposite to that in 4-mm group (287.62±22.63 vs 336.21±17.14), while no difference was detected for 6-mm group at 14 weeks after surgery (379.10±17.18 vs 381.94±18.17). Additionally, BV/TV of all the groups almost showed the same pattern as BMD (4-mm: 22.21±0,79 vs 16.68±1.52; 4-mm: 48.76±3.57 vs 46.09±3.25; 6-mm: 37.63±1.53 vs 36.21±3.74; 8-mm: 25.12±3.38 vs 32.51±2.03, respectively) (Fig 3C).

### Histological evaluation of bone

**Fig 4:**
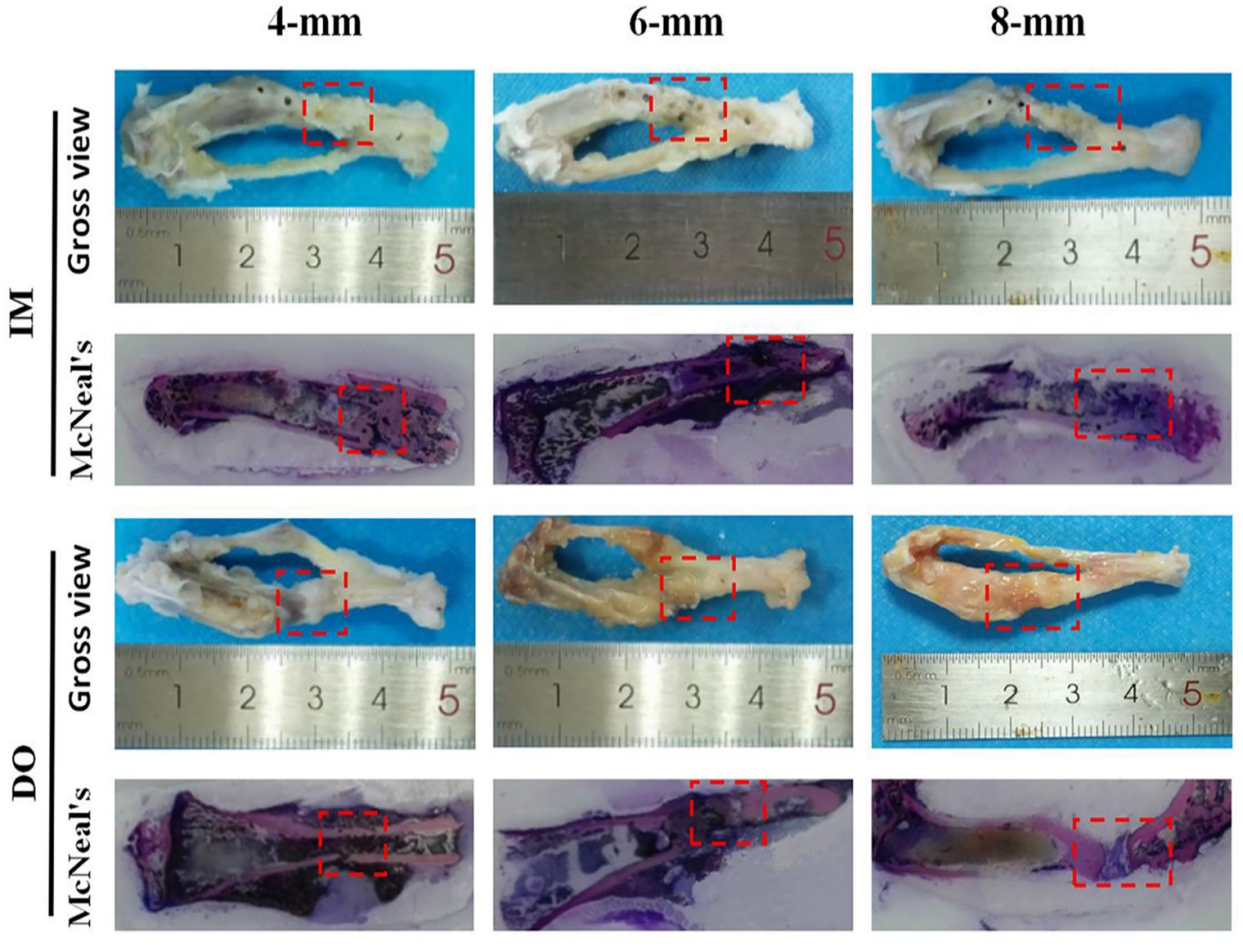
Overview and McNeal’s staining of representative samples harvested from 4-, 6- and 8-mm groups at 14 weeks after surgery. Undecalcified section were stained with McNeal’s. Mineralized bone tissue was stained pink and unmineralized tissue was light blue. Red dashed lines outline the defect regions.

**Fig 5:**
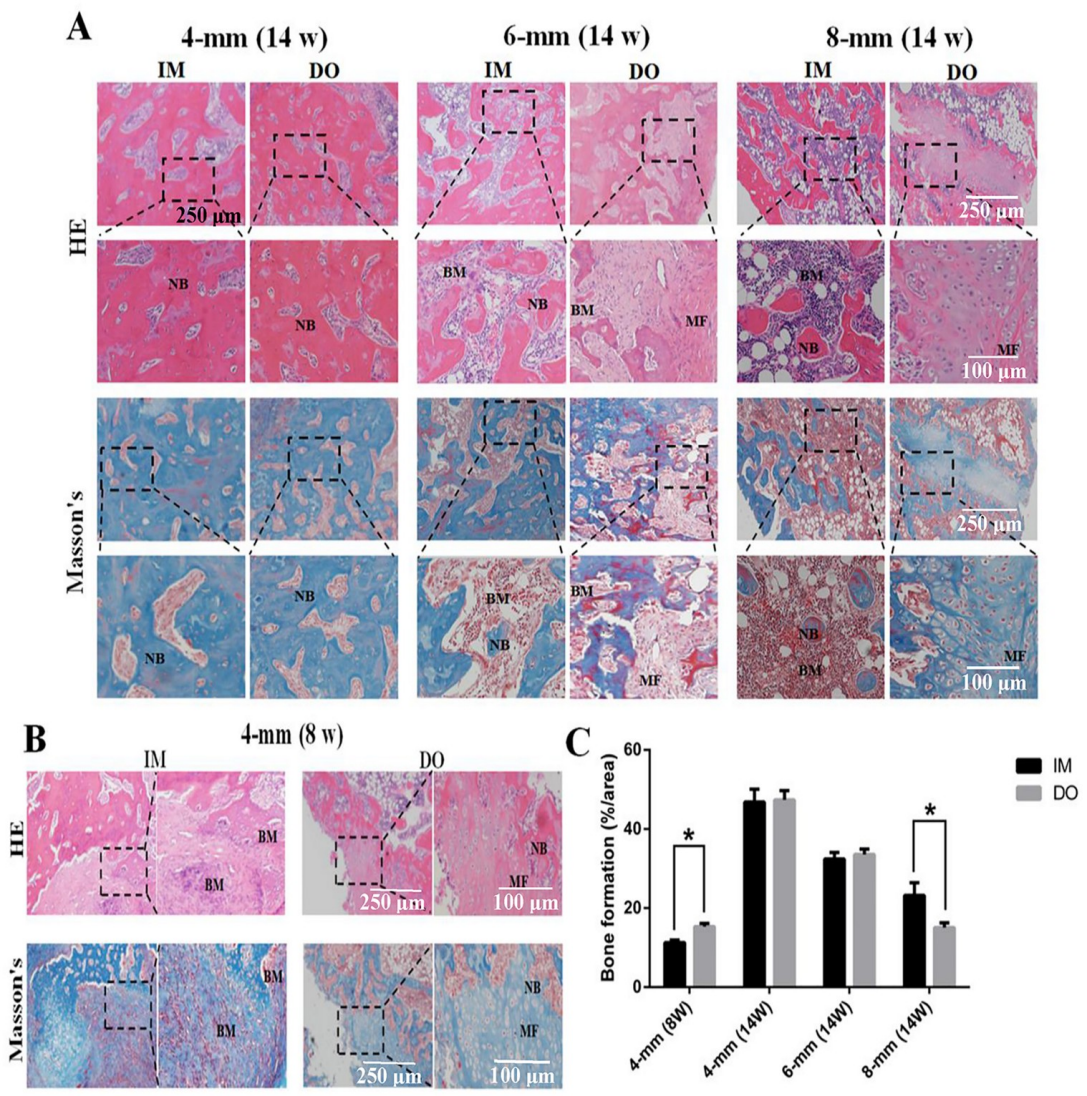
Histological analysis of newly-formed tissues within the defect regions of 4-, 6- and 8-mm groups. (A) Representative HE and Masson’s staining images of samples harvested at 14 weeks after surgery. The black boxes represent higher-magnification view of bone defect slices. Original magnification, 40×; higher magnification, 100×. (B) Representative histological images of samples harvested from the 4-mm group at 8 weeks postoperatively by HE and Masson’s staining. (C) Quantitative analysis of the bone formation fraction. NB: newly-formed bone; BM: bone marrow; MF: mineralized fiber-like tissue. (*P< 0.05).

**Fig 6:**
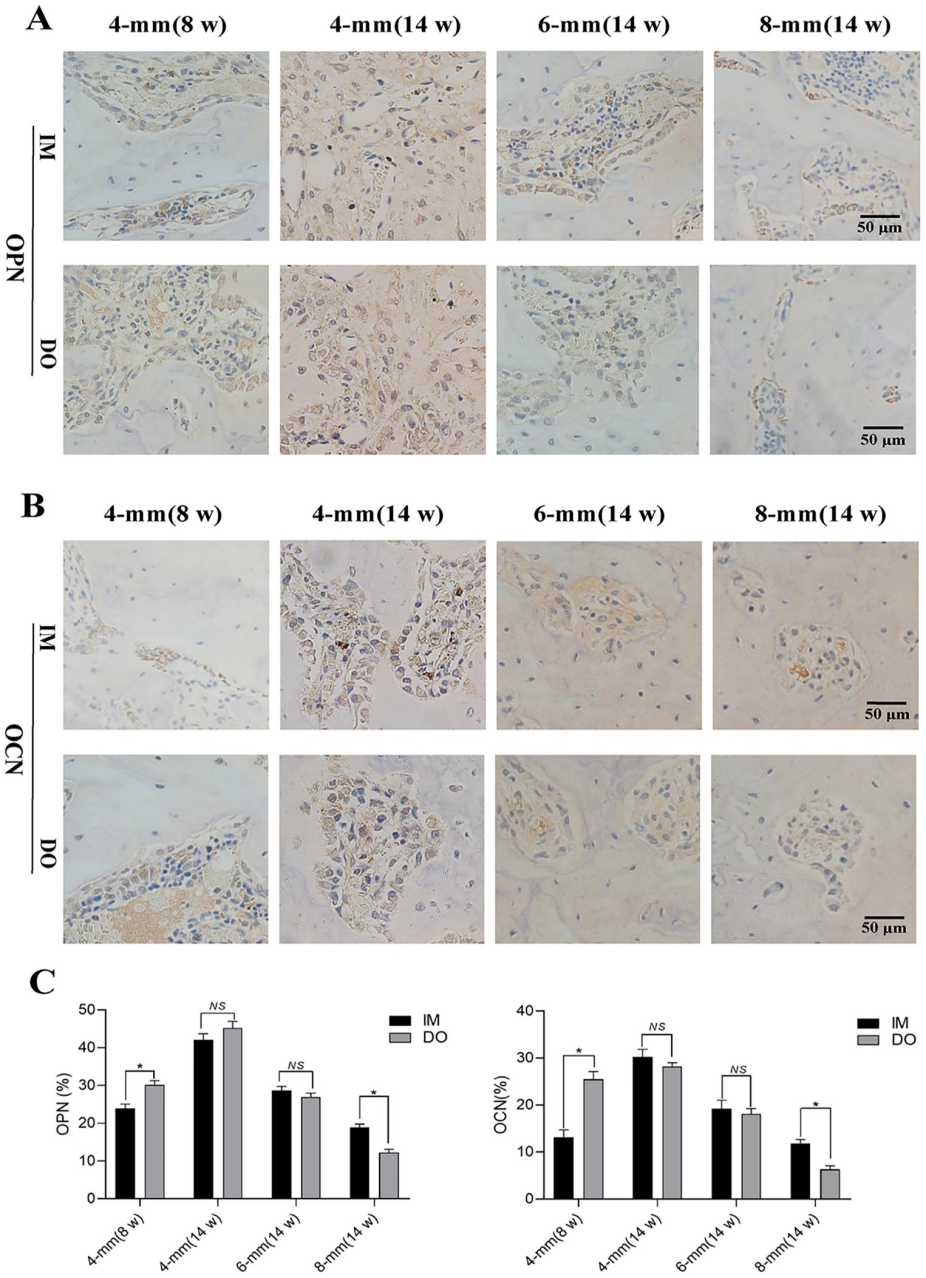
Immunohistochemical of OPN and OCN in newly-formed tissues within the defect regions of 4-, 6- and 8-mm groups. (A)-(B) Representative images of immunohistochemical results of OPN and OCN and (C) quantitative analysis of the positive cells in the bone defect regions. (*P< 0.05).

For bone repair, gross views and nondecalcificated sections of bone revealed that complete bony union inside defect regions have been achieved in 4-mm group at 14 weeks post-operation, while partial defect bridging occurred in 6-mm group and little bony union was observed in 8-mm group (Fig 4). Decalcified sections stained with HE and Masson’s further demonstrated that 4-mm group has experienced the complete defect healing and had the best bone connection and integration with both newly-formed bone tissue (NB) bridging the defects and bone marrow (BM) filling up the defect gap (Fig 5A). Of note, although there was no significant difference between IM- and DO-treated defects in 4-mm group at 14 weeks postoperatively, DO-treated defects earlier achieved mineralized callus and possessed more newly-formed bone than IM as early as the eighth week after surgery (Fig 5B); in 6-mm group, DO and IM showed almost similar capacity to generate new bone with no significant difference (Fig 5C); however, in 8-mm group, the results were reversed and IM displayed a higher proportion of new bone ingrowth into the defect gap, whereas only mineralized fiber-like tissue filled in the most area of the defect treated with DO (Fig 5B and 5C). The results of immunohistochemistry staining and quantitative analysis showed that the percentage of OPN-positive staining area of the defect region treated with DO (30.14±1.16) was higher than that of IM-treated defect region (23.94±1.14) as early as 8 weeks post operation for the 4-mm group, but no difference showed at 14 weeks (45.20±1.79 vs 42.08±1.62). However, the result in 8-mm group was opposite to that in 4-mm group (12.17±0.94 vs 18.90±0.88), while no difference was found for 6-mm group at 14 weeks after surgery (26.98±0.99 vs 28.64±1.14) (Fig 6A and 6C). Likewise, the expression of OCN of all the groups almost showed the almost same pattern as OPN (4-mm: 25.47±1.64 vs 13.16±1.54; 4-mm: 28.25±0.76 vs 30.21±1.63; 6-mm: 18.08±1.17 vs 19.22±1.82; 8-mm: 6.33±0.76 vs 11.86±0.79, respectively) (Fig 6B and 6C).

### Bone dynamic histomorphology evaluation

**Fig 7:**
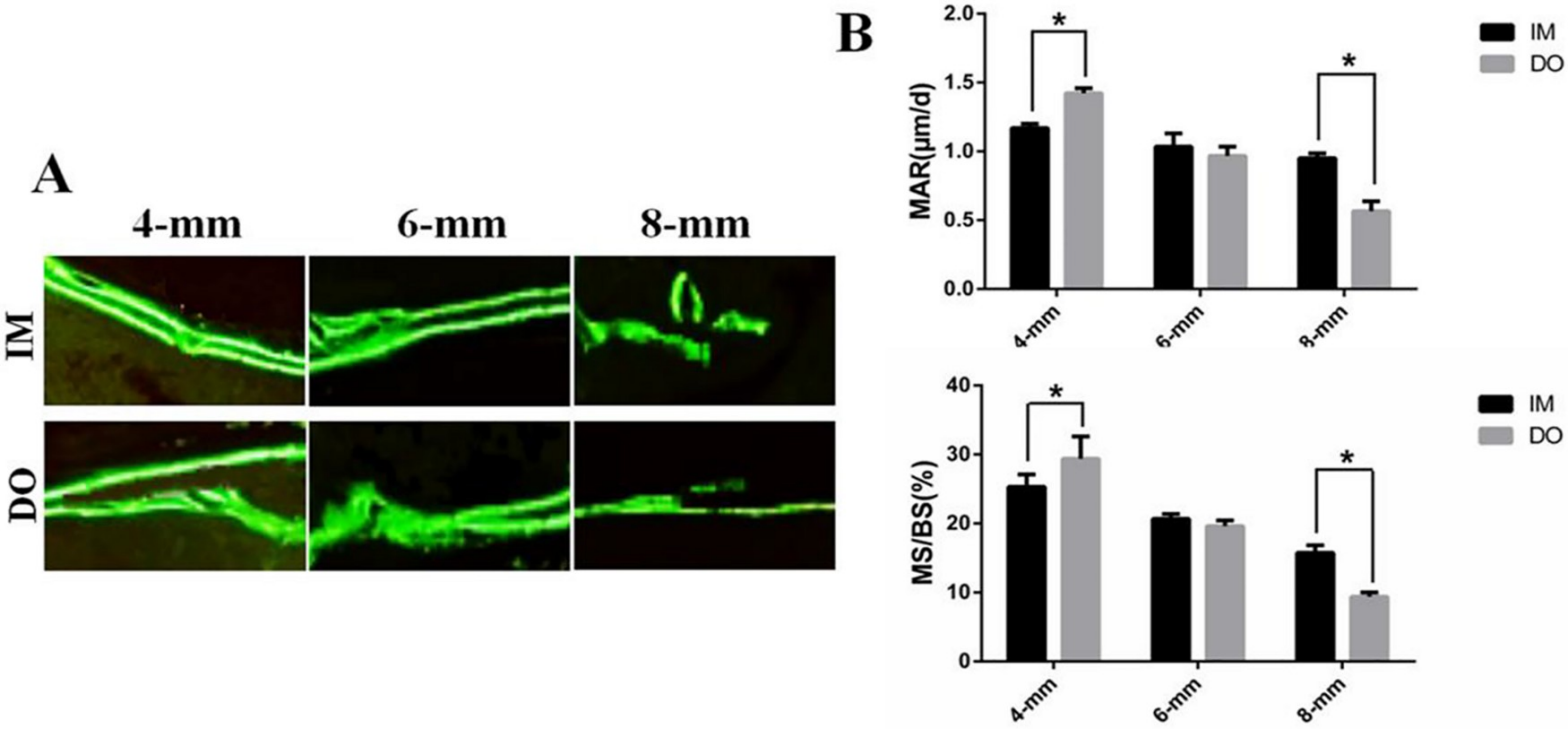
Dynamic bone formation illustrated by calcein double labeling. (A) Representative images of calcein double labeling of newly-formed bone. Original magnification, 200×. (B) Quantification of mineral apposition rate (MAR) and mineral surface versus bone surface (MS/BS). MAR (μm/day) = distance between labels/interlabel period. Horizontal interval of the lines represented the amount of newly-formed bone between the injection interphase. (*P< 0.05).

Generally, the result of MAR and MS/BS decreased as the defect size increased (Fig 7A). The results indicated that MAR and MS/BS of DO-treated defects (1.41±0.16 and 29.18±3.63) were found higher than those of IM-treated defects (1.17±0.12 and 25.69±1.46) in 4-mm group with a significant difference (Fig 7B). For 6-mm group, no significant differences were observed in MAR (0.95±0.20 vs 1.08±0.27) and MS/BS (18.81±0.93 vs 20.12±0.46), but in 8-mm group, DO resulted in lower MAR and MS/BS (0.58±0.19 and 9.56±0.71) than IM (0.92±0.14 and 14.33±1.02) and they differed significantly (Fig 7B).

### Histological evaluation of the membrane

**Fig 8:**
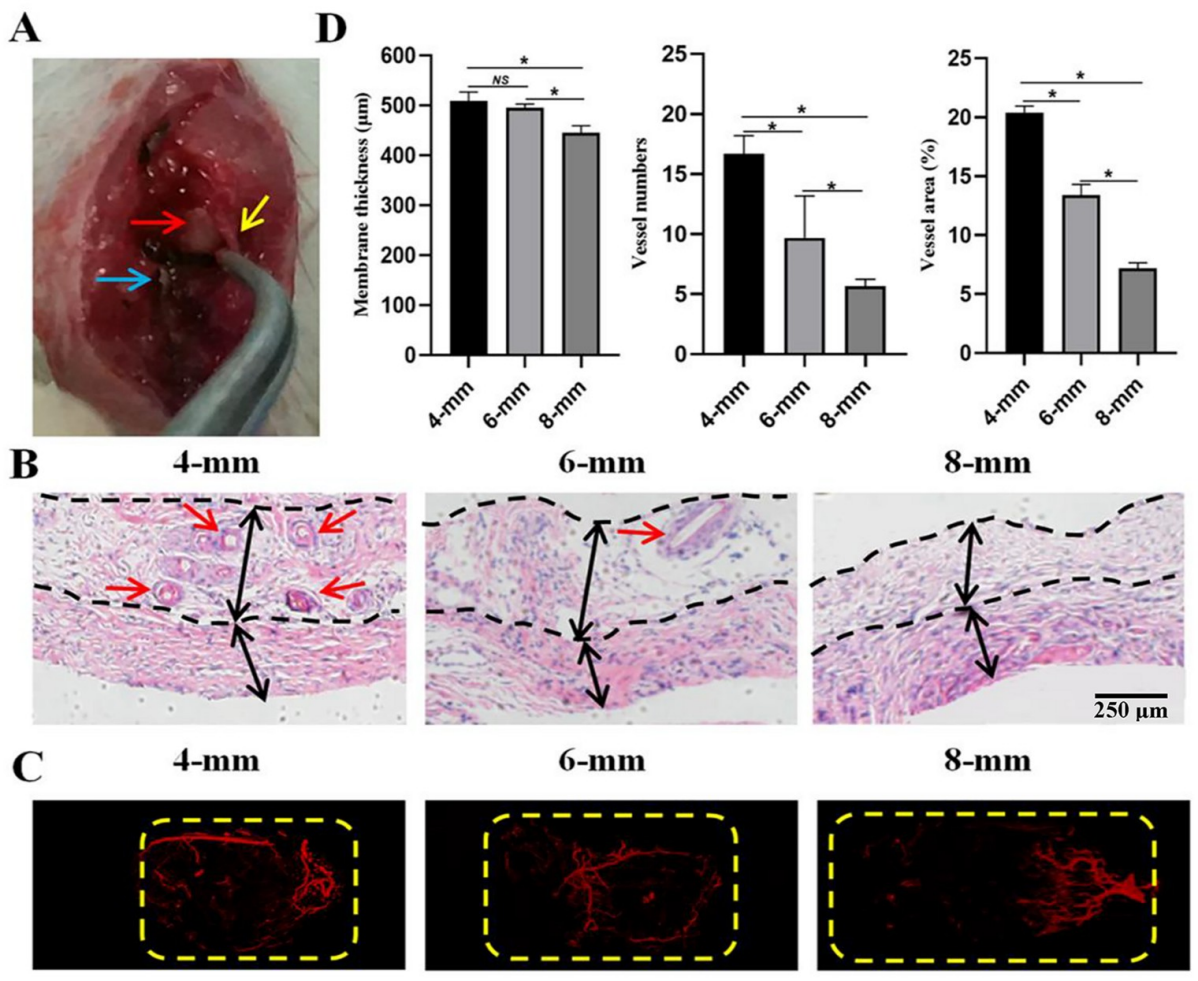
Histological analysis of the induced membranes within the defect regions of 4-, 6- and 8-mm groups. (A) Gross views of representative membrane samples of the three groups. Yellow arrow indicates the induced membrane, red arrow indicates the bone cement and blue arrow indicates the plate. (B) Representative histological (top panel) and angiographic (lower panel) images of the induced membranes by HE staining and three-dimensional reconstruction. Black arrows mark the dimensions of the fibrous (distal of the cement) and non-fibrous (proximal to the cement) layers of the induced membrane and interval between black dotted lines represent the membrane thickness. Red arrows indicate the vessels. Yellow dashed lines outline the defect regions. Original magnification, 40×. (C) Quantification of the membrane thickness and the vessel number and abundance inside the defect regions. (*P< 0.05; NS: not significant).

**Fig 9:**
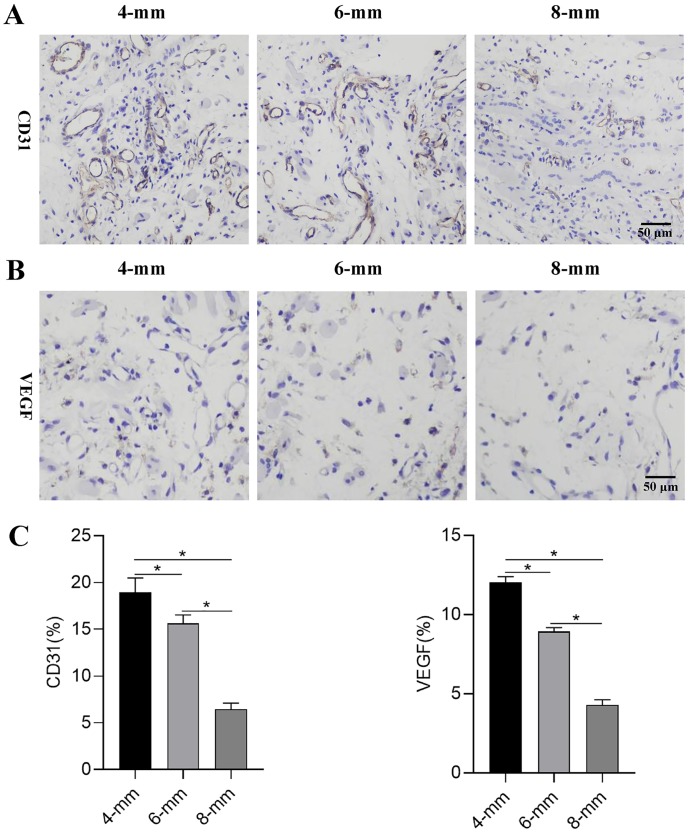
Immunohistochemical of CD31 and VEGF in the membrane tissues of 4-, 6- and 8-mm groups. (A)-(B) Representative images of immunohistochemical results of CD31 and VEGF and (C) quantitative analysis of the positive cells in the membrane tissues. (*P< 0.05).

For the induced membrane, gross observation revealed a vascularized tissue with histological properties similar to those of synovial tissue (Fig 8A) and the HE results demonstrated the presence of vasculature (Fig 8B). As shown in Fig 8B, the induced membrane thickness gradually decreased as the defect size increased. The membrane thickness of 8-mm group (436.43±18.65) was significantly thinner than those of 4-mm (510.27±20.36) and 6-mm (482.49±9.13) groups (Fig 8D). Besides, the vessel numbers (16.67±1.53; 9.67±3.51; 5.67±0.58, respectively) and abundance (20.09±2.18; 13.68±2.93; 7.11±1.93, respectively) obviously decreased as the defect size increased and significant differences were found among the groups (Fig 8D). Especially for the 8-mm group, distal to the defect area, the corresponding region in the membrane barely showed the vascular network (Fig 8C and 8D). The results of immunohistochemistry staining for CD31 (Fig 9A) and VEGF (Fig 9B) further confirmed that angiogenesis of the formed membranes obviously decreased as the defect size increased. The expressions of CD31 and VEGF in 8-mm group (6.45±0.66 and 4.30±0.32) was significantly lower than those of 4-mm (18.97±1.51 and 12.04±036) and 6-mm (15.63±0.90 and 8.93±0.25) groups and significant differences were observed among the groups (Fig 9C).

## Discussion

Many factors can affect the plans of therapies for SBDs such as soft tissue condition, infection, cost and even the surgeon’s preferences or experiences [23–25]. One of the most fundamental aspects is the defect size. To the best of our knowledge, this is the first study to report the differences between IM and DO in treating different sizes of bone defects and to determine the most appropriate defect size for IM or DO.

In this study, we firstly successfully constructed rat models of 4-, 6-and 8-mm long mid-diaphyseal defects on bilateral tibias using DO and IM techniques, respectively. Based on the definition and characteristics of SBDs [6, 8], the 4-, 6- and 8-mm rat tibia defects represent small-sized, moderate-sized and over-sized SBDs, respectively. From our study results, DO were found to achieve better bone repair than IM in small-sized SBDs, with higher expressions of OPN and OCN, faster bone formation rate, shorter treatment duration, higher bone formation fraction and earlier bony connection, but IM could outperform DO in bone repair capacity for over-sized SBDs, which coincides with the report by O’Malley et al. [32] who reported a successful reconstitution of a 17 cm diaphyseal defect in the tibia using the Masquelet technique. In addition, defects up to 25 cm have also been reported to have fully consolidated with the masquelet technique within 12 months [10], which confirms the advantages of IM in treating larger-scale bone defects.

Although numerous studies have been made to investigate the pros and cons of IM and DO for SBDs [25, 33], as far as we know, it is the first time to provide references for selecting IM or DO for SBDs from the perspective of the bone defect size. The differences between IM and DO in treating different sizes of bone defects may be partially due to the difference in osteogenic patterns and characteristics of the two. Ossification is usually classified into two types: intramembranous (direct) and endochondral (indirect) [34]. There is no pre-existent cartilage model and new bone is directly formed by differentiated osteoblasts in the mode of intramembranous ossification, while in endochondral ossification, a highly ordered structure of resting, proliferating, hypertrophic, and calcifying cartilage is first formed by differentiating chondrocytes, and then the calcified cartilage matrix is invaded by capillaries and new bone is laid down by osteoblasts in the space previously occupied by hypertrophic chondrocytes, which suggests more complex processes with more prolonged osteogenesis period exist in endochondral ossification than in intramembranous ossification[35].

For IM, the osteogenesis way of IM is reported to be similar to intramembranous osteogenesis [36–38] and bone formation could occur synchronously over the whole region in direct contact with the induced membrane, which subsequently contributes to faster healing [38]. Although the defect length increased, the entire region covered by the induced membrane still achieved synchronous bone formation as long as the corresponding size of induced membrane can be formed at the defect area. By contrast, the osteogenic mode of DO is composed of both intramembranous and endochondral ossification [34, 39, 40]. Growing evidence have suggested that typical endochondral ossification was prominent in the early stage of distraction, while intramembraneous ossification became the predominant mechanism of osteogenesis at later stages [34, 41, 42], which indicates that more complex steps of osteogenesis occur during DO process. As previous studies described [43–45], during the consolidation phase, the callus formation or bone mineralization mainly started from the distal and proximal ends of the defect gap, gradually moved to the central area, and finally accomplished an bony connection, which was more obvious in the larger defect gap model. The size of the bone defect gap determines the duration of distraction phase. Consequently, the time to healing is dependent on the size of the defect gap and the larger the defect gap is, the more time it will take to accomplish the bridging between the defect gap ends, which is confirmed by X-ray and micro-CT results in this study.

Besides, the osteogenesis way is subjected to the local biomechanical properties at the injured region, which indicates that local biomechanical properties in DO and IM models might be another reason for the findings. According to Wolff’s law, mechanical stimulation plays an important role in modeling and remodeling of bone. Reasonable structural properties of the fixation can spread the force evenly across the entire shaft of the bone. However, subtle changes in mechanical conditions may affect the stability of a device that is subjected to loading, and subsequently exert influence on the osteogenesis way. As reported previously [46, 47], bone healing mechanisms were different and dependent on the fracture stability and the rigidity of the device used. Rigid fixation and stable environments at bone injured site contributed to direct (intramembraneous) ossification, while indirect (endochondral) ossification was induced because of the less rigid fixation and unstable environments. In this study, the differences in mechanical properties of the internal plate and external fixator such as anti-compression, anti-bending and anti-torsion could cause different biomechanical environments, which may affect the osteogenic patterns. In 4-mm defect group, the duration of distraction was relatively short and the circular external provides sufficient stability for bone injured region, which ensured intramembranous ossification as predominant mechanism of osteogenesis. However, as the bone defect gap extended, the distraction duration gradually increased in 6- and 8-mm defect groups. Moreover, local bone resorption around the long-time loading pin and subsequent loosening would cause less rigid fixation over time, which meant endochondral ossification took up most of the process of DO and spent more time on bone healing. The present study results were in accordance with the report by Wu et al, who reported the longer period was required for bone repair when external fixation with less rigidity was used [48].

In addition, from the perspective of cellular events, some important healing parameters such as cell migration, diffusion and solute transport could be affected by the length-scale of the injured or defect site [29]. And especially for mesenchymal stem cells (MSCs), they exert exceptional differentiation capabilities toward osteo-angiogenesis and play an important role in bone healing and regeneration. For one thing, such proteins as OPN, involved in osteogenic differentiation, could be produced by MSCs. For another, the expression of the receptors of VEGF and PDGF in surrounding mesenchymal cells involves MSCs [49]. More importantly, gradual and continuous strain could stimulate the migration and transport of MSCs to the bone injured site and subsequently contribute to bone healing and regeneration during DO process, while the presence and concentration of MSCs in induced membranes was found to be associated with the biological activity of the membranes and MSCs could support bone healing during application of IM technique in previous studies[27, 37], which might represent a new perspective to explain the effects of different defect sizes on bone healing of DO and IM. As is well known, the bone repair capacity of IM largely depends on the bioactivities of the formed membrane tissue. However, it is not clear whether the bone defect sizes affect the bioactivities of membranes. It is noteworthy that the formed membranes in our rat model were found to be highly vascularized, which is in correspondence with that reported by Masquelet et al. However, the membrane formed in 8-mm group was obviously thinner than the other two groups, with lower concentration of CD31 and VEGF, lower vessel number and abundance. These findings demonstrated that the structure and activity of the induced membrane were subjected to the defect size, which to some extent accords with the previous study reporting that length-scale of injured site can certainly affect some important healing parameters such as cell migration, diffusion, solute transport and subsequent repair time [29]. According to Masquelet et al., the formation of an induced membrane around an implant needs to be covered by healthy soft tissues such as muscles to stimulate foreign body reaction and bone grafts should be covered by soft tissues to be revascularized. However, it is well known that most muscle tendons instead of muscle bellies are present at the distal site of the tibia. There may be not enough tissues to cover the distal site of the PMMA cement to induce the membrane formation, when the bone defect size is rather large, which indicates that advantages of IM over DO in larger bone defects are based on good soft tissue conditions. Especially in clinical practice, the induced membrane formation and subsequent bone repair could be impaired in bone defects combined with soft tissue injury or wound infection.

Although our study provided a new perspective on the choice between DO and IM for the treatment of SBDs, two limitations should be considered when interpreting our results. First, as a preclinical study, this work temporarily did not elucidate how the defect sizes in the rat model correspond with the human situations and subsequently the findings in the rat model may not be extrapolated directly to human conditions for the time being. However, many discoveries that went on to greatly impact clinical practice were first observed and studied in animal models [50]. Any major procedural change or adjuvant therapy should be vetted in a animal model prior to clinical implementation, and rodent use is a good way to screen these procedures and investigate basic biology, which implies that the study is still of great significance to further clinical practice. Second, the rats used in this study were relatively young. The rats with the age of 20 weeks old are physiologically similar to young adult humans with the age of 20 to 30 years old [51]. Many pathophysiological processes alter as an organism ages, which is especially true for bone development and repair [36]. It’s unknown whether similar results would be obtained using different aged animals in this study. Therefore, aging is an important factor that should be further studied in the future.

Taken together, our results reveal that the therapeutic effects of IM and DO may be subjected to sizes of bone defects and the best treatment size of defects is different between the two. For small-sized SBDs, DO is more suitable and efficient than IM with faster bone formation rate, shorter treatment duration and earlier bony connection, but conversely, IM outperformed DO in bone repair capacity for over-sized SBDs, while DO and IM seem to possess the similar bone repair capacity in the case of moderate-sized SBDs, which may have some implications for our clinical practice and at least would provide references for further clinical research.

## Supporting information

S1 Table Raw data for main figures. (A) Radiographic scores of Fig 2B, (B) BMD and BV/TV of Fig 3C, (C) Bone formation fraction of Fig 5C, (D) OPN and CON positive staining area percentage of Fig 6C, (E) MAR and MS/BS of Fig 7B, (F) The membrane thickness, vessel number and abundance of Fig 8D, (G) CD31 and VEGF positive staining area percentage of Fig 9C.
(DOC)
