# Supplementary material for: Comparison between the induced membrane technique and distraction osteogenesis in treating segmental bone defects: An experimental study in a rat model
Source: PLoS One. 2019 Dec 20;14(12):e0226839. doi: 10.1371/journal.pone.0226839 (PMC6924672; doi:10.1371/journal.pone.0226839)
Supplement: S1 Table — (A) Radiographic scores of Fig 2B, (B) BMD and BV/TV of Fig 3C, (C) Bone formation fraction of Fig 5C, (D) OPN and CON positive staining area percentage of Fig 6C, (E) MAR and MS/BS of Fig 7B, (F) The membrane thickness, vessel number and abundance of Fig 8D, (G) CD31 and VEGF positive staining area percentage of Fig 9C. (DOC) [file pone.0226839.s001.doc]

**Supporting Information**

| **A** | | | | | | | | | | |
| --- | --- | --- | --- | --- | --- | --- | --- | --- | --- | --- |
| **Group** | | **Radiographic scores of sample 1** | | | **Radiographic scores of sample 2** | | | **Radiographic scores of sample 3** | | |
|  |  | **Surgeon 1** | **Surgeon 2** | **Surgeon 3** | **Surgeon 1** | **Surgeon 2** | **Surgeon 3** | **Surgeon 1** | **Surgeon 2** | **Surgeon 3** |
| 4-mm(8 w) | IM | 3 | 3 | 4 | 3 | 4 | 3 | 4 | 4 | 4 |
|  | DO | 4 | 5 | 5 | 4 | 5 | 4 | 5 | 4 | 5 |
| 4-mm(12 w) | IM | 8 | 8 | 9 | 8 | 8 | 8 | 9 | 9 | 8 |
|  | DO | 8 | 9 | 9 | 9 | 9 | 8 | 10 | 9 | 9 |
| 4-mm(14 w) | IM | 12 | 11 | 12 | 12 | 12 | 12 | 12 | 11 | 11 |
|  | DO | 12 | 13 | 11 | 12 | 13 | 13 | 12 | 12 | 11 |
| 6-mm(8 w) | IM | 3 | 2 | 4 | 2 | 3 | 4 | 3 | 3 | 3 |
|  | DO | 3 | 3 | 2 | 3 | 4 | 2 | 3 | 3 | 4 |
| 6-mm(12 w) | IM | 6 | 8 | 6 | 7 | 7 | 8 | 7 | 7 | 6 |
|  | DO | 8 | 6 | 7 | 8 | 8 | 7 | 7 | 6 | 7 |
| 6-mm(14 w) | IM | 10 | 10 | 9 | 10 | 9 | 9 | 9 | 10 | 10 |
|  | DO | 9 | 10 | 9 | 9 | 9 | 9 | 10 | 10 | 10 |
| 8-mm(8 w) | IM | 3 | 3 | 2 | 3 | 2 | 3 | 3 | 3 | 3 |
|  | DO | 1 | 1 | 3 | 1 | 1 | 2 | 1 | 1 | 1 |
| 8-mm(12 w) | IM | 5 | 4 | 6 | 5 | 5 | 6 | 5 | 6 | 4 |
|  | DO | 3 | 4 | 2 | 3 | 2 | 4 | 3 | 3 | 3 |
| 8-mm(14 w) | IM | 9 | 8 | 10 | 9 | 8 | 9 | 9 | 9 | 8 |
|  | DO | 8 | 6 | 7 | 6 | 7 | 7 | 7 | 6 | 7 |

| **B** | | | | | | | | | | | | | |
| --- | --- | --- | --- | --- | --- | --- | --- | --- | --- | --- | --- | --- | --- |
| **Group** | | **BMD (mg/cm^3^)** | | | | | | **BV/TV (%)** | | | | | |
|  |  | **Sample 1** | | **Sample 2** | | **Sample 3** | | **Sample 1** | | **Sample 2** | | **Sample 3** | |
| 4-mm  (8 w) | IM | | 242.86 | | 221.94 | | 263.78 | | 16.68 | | 15.16 | | 18.20 |
|  | DO | | 271.22 | | 285.71 | | 300.20 | | 23.00 | | 22.21 | | 21.42 |
| 4-mm  (14 w) | IM | | 424.90 | | 403.68 | | 414.29 | | 49.34 | | 42.84 | | 46.09 |
|  | DO | | 446.52 | | 432.58 | | 418.64 | | 52.33 | | 45.19 | | 48.76 |
| 6-mm  (14 w) | IM | | 398.21 | | 362.33 | | 385.27 | | 32.47 | | 36.21 | | 39.95 |
|  | DO | | 379.10 | | 361.92 | | 396.28 | | 37.63 | | 36.10 | | 39.16 |
| 8-mm  (14 w) | IM | | 336.24 | | 319.05 | | 353.33 | | 32.51 | | 30.48 | | 34.54 |
|  | DO | | 310.25 | | 264.99 | | 287.62 | | 21.74 | | 25.12 | | 28.50 |

| **C** | | | | |
| --- | --- | --- | --- | --- |
| **Group** | | **Bone formation fraction (%)** | | |
|  |  | **Sample 1** | **Sample 2** | **Sample 3** |
| 4-mm  (8 w) | IM | 12.33 | 12.75 | 11.88 |
|  | DO | 16.60 | 15.99 | 17.21 |
| 4-mm  (14 w) | IM | 47.13 | 50.48 | 43.78 |
|  | DO | 47.80 | 44.58 | 51.02 |
| 6-mm  (14 w) | IM | 32.29 | 30.47 | 34.11 |
|  | DO | 34.10 | 35.73 | 32.47 |
| 8-mm  (14 w) | IM | 24.18 | 27.43 | 20.93 |
|  | DO | 18.04 | 16.92 | 19.16 |

| **D** | | | | | |
| --- | --- | --- | --- | --- | --- |
| **Group** | | **OPN(%)** | | **OCN (%)** | |
| 4-mm  (8 w) | IM | | 23.94±1.14 | | 13.16±1.54 |
|  | DO | | 30.14±1.16 | | 25.47±1.64 |
| 4-mm  (14 w) | IM | | 42.08±1.62 | | 30.21±1.63 |
|  | DO | | 45.20±1.79 | | 28.25±0.76 |
| 6-mm  (14 w) | IM | | 28.64±1.14 | | 19.22±1.82 |
|  | DO | | 26.98±0.99 | | 18.08±1.17 |
| 8-mm  (14 w) | IM | | 18.90±0.88 | | 11.86±0.79 |
|  | DO | | 12.17±0.94 | | 6.33±0.76 |

| **E** | | | | | | | |
| --- | --- | --- | --- | --- | --- | --- | --- |
| **Group** | | **MAR (μm/d)** | | | **MS/BS (%)** | | |
|  |  | **Sample 1** | **Sample 2** | **Sample 3** | **Sample 1** | **Sample 2** | **Sample 3** |
| 4-mm | IM | 1.17 | 1.05 | 1.29 | 25.69 | 24.23 | 27.15 |
|  | DO | 1.25 | 1.41 | 1.57 | 26.55 | 32.81 | 29.18 |
| 6-mm | IM | 1.08 | 1.35 | 0.81 | 20.12 | 19.66 | 20.58 |
|  | DO | 1.15 | 0.75 | 0.95 | 19.74 | 18.81 | 17.88 |
| 8-mm | IM | 1.06 | 0.78 | 0.92 | 13.31 | 15.35 | 14.33 |
|  | DO | 0.39 | 0.58 | 0.77 | 8.85 | 9.56 | 10.27 |

| **F** | | | | | | | | | |
| --- | --- | --- | --- | --- | --- | --- | --- | --- | --- |
| **Group** | **Membrane thickness (μm)** | | | **Vessel number** | | | **Vessel area (%)** | | |
|  | **Sample 1** | **Sample 2** | **Sample 3** | **Sample 1** | **Sample 2** | **Sample 3** | **Sample 1** | **Sample 2** | **Sample 3** |
| 4-mm | 530.63 | 510.27 | 489.91 | 15 | 18 | 17 | 20.09 | 22.27 | 17.91 |
| 6-mm | 473.36 | 482.49 | 491.62 | 10 | 6 | 13 | 10.75 | 16.61 | 13.68 |
| 8-mm | 436.43 | 417.78 | 455.08 | 5 | 6 | 6 | 5.18 | 7.11 | 9.04 |

| **G** | | | | |
| --- | --- | --- | --- | --- |
| **Group** | **CD31(%)** | | **VEGF (%)** | |
| 4-mm | | 18.97±1.51 | | 12.04±036 |
| 6-mm | | 15.63±0.90 | | 8.93±0.25 |
| 8-mm | | 6.45±0.66 | | 4.30±0.32 |

**S1 Table. Raw data for main figures.** (A) Radiographic scores of Fig 2B, (B) BMD and BV/TV of Fig 3C, (C) Bone formation fraction of Fig 5C, (D) OPN and CON positive staining area percentage of Fig 6C, (E) MAR and MS/BS of Fig 7B, (F) The membrane thickness, vessel number and abundance of Fig 8D, (G) CD31 and VEGF positive staining area percentage of Fig 9C.
